# Cierre percutáneo de vena vertical, posterior a la reparación de conexión anómala pulmonar total supracardiaca, usando oclusor para defecto del septum atrial. Reporte de caso

**DOI:** 10.47487/apcyccv.v4i4.319

**Published:** 2023-12-27

**Authors:** Alex Catalán Cabrera, Karen Condori Alvino

**Affiliations:** 1 Área de Cateterismo Cardiaco Pediátrico, Instituto Nacional de Salud del Niño de San Borja, Lima, Perú. Área de Cateterismo Cardiaco Pediátrico Instituto Nacional de Salud del Niño de San Borja Lima Perú

**Keywords:** Venas Pulmonares, Dispositivo Oclusor Septal, Cardiopatía Congénita, Pulmonary vein, Septal Occluder Device, congenital heart disease

## Abstract

En los pacientes operados de conexión venosa pulmonar anómala total supracardiaca (CVPAT-SC) el no ligar la vena vertical (VV) de manera rutinaria ayuda a mantener una mayor estabilidad hemodinámica en el posoperatorio y en muchos de los casos se logrará un cierre espontáneo. Sin embargo, si permanece patente la VV, condiciona a tener un cortocircuito pretricuspídeo con hiperflujo pulmonar significativo, lo que en la mayoría de los casos requiere el cierre quirúrgico o percutáneo. Se presenta el caso de un paciente posoperado de CVPAT-SC no obstructiva con VV patente, en el que se realizó el cierre vía percutánea utilizando oclusor para defecto del septum interatrial.

## Abstract (en)

In patients operated on for total supracardiac anomalous pulmonary venous connection (TAPVC-SC), not ligating the vertical vein (VV) routinely helps to maintain greater hemodynamic stability in the postoperative period, and in many cases, spontaneous closure will be achieved. However, if the VV remains patent, it leads to a pre-tricuspid shunt with significant pulmonary hyperflow, requiring surgical or percutaneous closure. We present the case of a post-operated patient for non-obstructive TAPVC-SC with patent VV, in whom percutaneous closure was performed using an atrial septal Occluder.

## Introducción

La conexión venosa anómala pulmonar total (CVAPT) es definida como una anomalía en la cual las venas pulmonares no tienen conexión con el atrio izquierdo, donde las venas están directamente conectadas a una de las venas sistémicas o pueden drenar directamente al atrio derecho ^1^.

La frecuencia de CVAPT es entre 1-1,5% de todas las cardiopatías congénitas y puede estar asociada a otras anomalías cardiacas, especialmente el síndrome de heterotaxias ^2^.

Los síntomas en estos pacientes pueden variar, y todo va a depender si es obstructiva o no obstructiva. Los pacientes con CVAPT no obstructiva son usualmente asintomáticos al nacimiento. Sin embargo, a las primeras semanas de vida pueden presentar cardiomegalia, disnea al esfuerzo, leve cianosis y dificultad respiratoria; mientras que los niños con CVAPT obstructiva presentarán problemas respiratorios, hipoxia, hipertensión pulmonar, y rápida progresión a falla cardiorrespiratoria ^1^. 

La reparación de la CVAPT supracardiaca ha tenido algunas modificaciones, y muchos cirujanos prefieren ligar la vena vertical (VV) para prevenir cortocircuitos residuales de izquierda a derecha. Sin embargo, con cámaras izquierdas pequeñas y sin adecuada *compliance* de la aurícula izquierda, el no ligar la vena vertical puede mejorar la supervivencia ya que provee una descompresión transitoria del lado derecho del corazón para las crisis de hipertensión pulmonar en el posoperatorio inmediato ^3^^,^^4^. Empero, esta VV puede permanecer permeable durante el tiempo sin cierre espontaneo, lo cual va a provocar cortocircuito de izquierda a derecha, siendo necesario en muchas ocasiones el cierre quirúrgico o percutáneo de este defecto residual.

## Reporte de caso

Se reporta el caso de un varón de 12 años, procedente de Lima - Perú, quien tenía como antecedente CVAPT no obstructiva más comunicación interauricular diagnosticada a los 6 meses de edad, sin historia de hospitalizaciones u otro antecedente de importancia.

El paciente fue referido a nuestra institución por primera vez a los 9 años, presentando disnea y leve cianosis a grandes esfuerzos, sin historia de hospitalizaciones previas, saturación de oxígeno 96%. En la radiografía de tórax se observó dilatación de cavidades derechas con la característica de «muñeco de nieve». En la ecocardiografía se evidenció dilatación de cavidades derechas, comunicación interauricular de 10 mm de diámetro; no se observó llegada de venas pulmonares a la aurícula izquierda, por el contrario, se evidenció que el flujo de las venas pulmonares se continúa con una VV que se conecta hacia arriba con la vena innominada, no se detectó gradiente obstructivo en dicha vena. La presión pulmonar estimada por regurgitación tricuspidea fue de 30 mmHg.

Con estos datos se confirmó el diagnóstico de CVAPT supracardiaca no obstructiva, por lo que se inicia terapia médica con diuréticos: furosemida 1 mg/kg cada 12 h y espironolactona 1 mg/kg cada 24 h. Además, luego de analizar el caso, se decidió corrección quirúrgica de dicha malformación.

Dado el tiempo de presentación de la cardiopatía fue sometido a cateterismo cardiaco previo a cirugía para medir presiones cardiacas; se halló hipertensión pulmonar leve; presión diastólica final de ventrículo izquierdo (PDFVI) 10 mmHg; presión diastólica final de ventrículo derecho 8 mmHg; resistencia vascular pulmonar (RVP) 1,8 UW/m^2^, RVP/RVS 0,16. Con estos valores fue sometido a cirugía cardiaca correctiva con corrección retroauricular más cierre de comunicación interauricular con parche pericardio autólogo, dejando la VV permeable; con tiempo de circulación extracorpórea de 130 min y tiempo de clampaje de 84 min. En el posoperatorio no se presentó crisis de hipertensión pulmonar, pero sí *flutter* atípico revirtiendo a ritmo sinusal con cardioversión eléctrica; permaneció en la unidad posoperatoria cardiovascular por 7 días, pasando luego a hospitalización cardiovascular, para finalmente ser dado de alta luego de 3 días (al décimo primer día del posoperatorio).

**Figura 1:**
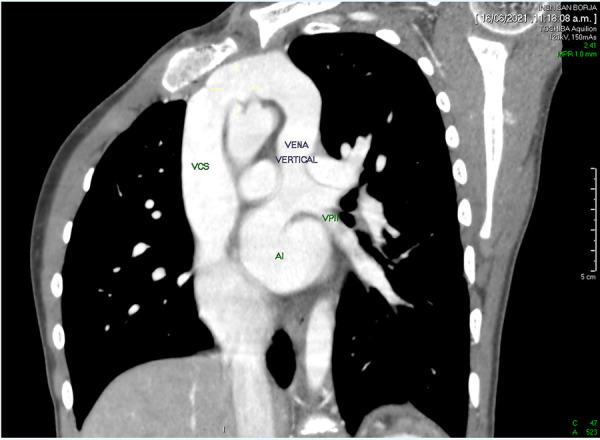
Tomografía contrastada, muestra la patencia de vena vertical poscorrección quirúrgica de CVAPT. VCS: vena cava superior. AI: aurícula izquierda. VPII: vena pulmonar inferior izquierda.

**Figura 2:**
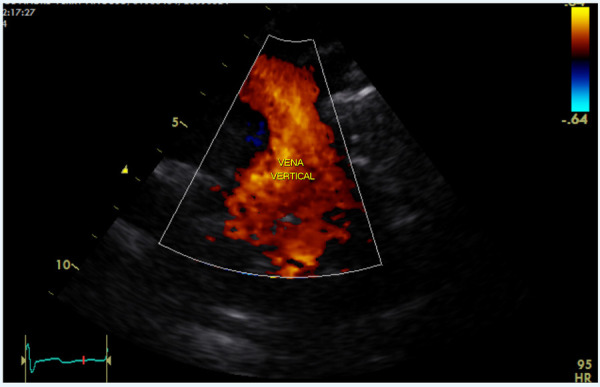
Ecocardiograma vista supraesternal con persistencia de vena vertica.

En los controles ambulatorios, el paciente persistía con disnea CF II y saturación de oxígeno 96%. En controles ecocardiográficos seriados se mantuvo la dilatación de cavidades derechas, llegada de venas pulmonares a aurícula izquierda sin estenosis y VV permeable, sin gradiente obstructivo, **(**Figura 1**)** no evidenciando proceso de cierre espontáneo. Se complementó estudio con tomografía cardiaca contrastada, la cual confirma la patencia de la VV **(**Figura 2**).**

**Figura 3:**
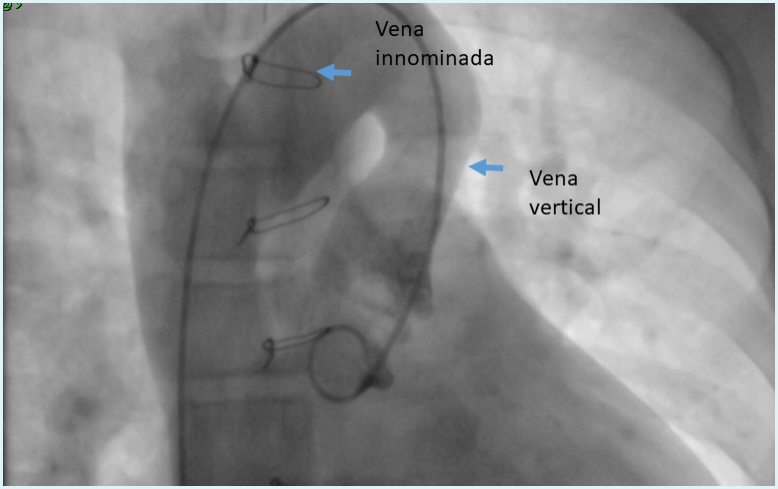
Angiografía, en proyección frontal la VV midió 17 mm y en proyección lateral midió 22 mm.

**Figura 4:**
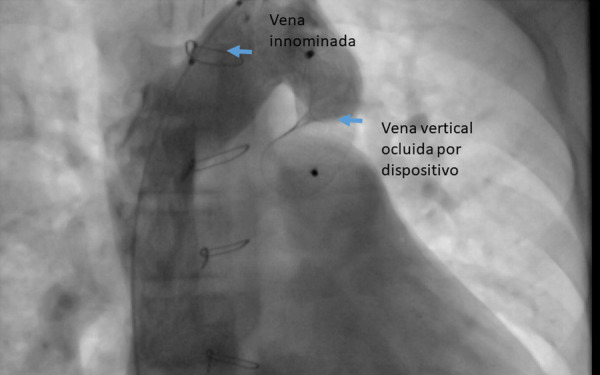
*Amplatzer Septal Occluder* N.°24 dejado posicionado en vena vertical.

Luego de 2 años de la cirugía, debido a la persistencia de los síntomas y ante la evidencia de la patencia de la VV, se decide realizar cierre percutáneo de la misma. A través de acceso en vena femoral derecha e izquierda, se realizó estudio hemodinámico previo, encontrando QP/QS de 2,9; RVP 0,51 UW/m^2^, PDFVI: 9 mmHg, presión arterial pulmonar (PAP) media: 11 mmHg, presión de aurícula izquierda:12 mmHg. Con estos valores se ingresó catéter coronario derecho 5 Fr y guía hidrofílica 0,035” x 150 cm VCS- Vena innominada - vena vertical. Se realizó angiografía en proyección 0°/0° y RAO 90°/0°, se procedió a realizar mediciones de VV que en proyección frontal mide 17 mm y proyección lateral mide 22 **(**Figura 3**,** Video 1**);** se intercambió guía hidrofílica 0.035” x 180 cm por guía extrasoporte 0,035” x 260 cm y se dejó posicionada en vena pulmonar superior derecha. Se ingresó introductor largo 9 Fr para un oclusor de defecto interatrial (Amplatzer Septal Occluder N.° 24), el cual queda posicionado en vena vertical. Se realizó angiografías de control a través catéter Pigtail 5 Fr ingresado por el acceso venoso femoral izquierdo, verificando que la confluencia de la yugular con la subclavia y la vena innominada esté libre procediendo a liberar el dispositivo **(**Figura 4**)**. El Video 2 muestra la VV debidamente ocluida.

El paciente fue dado de alta a las 24 h del procedimiento sin complicaciones mayores, saturación de oxígeno de 97%, la ecocardiografía al alta no mostró evidencia de flujo de la VV hacia vena innominada. Se continuó tratamiento médico con furosemida 1 mg/kg cada 24 h y espironolactona 1 mg/kg cada 24 h; medicación que luego de 6 meses de seguimiento se suspendió por la evolución clínica favorable.

## Discusión

La CVAPT es una cardiopatía poco frecuente en el cual las venas pulmonares no se conectan al atrio izquierdo, tiene una mortalidad del 50% hasta el tercer mes de vida y supervivencia al año sin intervención terapéutica del 20% ^3^; debe considerarse que de preferencia la corrección debe ser realizada al momento del diagnóstico, que idealmente se realiza en recién nacidos, y si se deja la vena vertical patente es posible la oclusión espontanea en el tiempo, no siendo el caso de nuestro paciente que presentó una evolución natural con corrección tardía del defecto.

La mayoría de CVPAT se caracterizan por la existencia de un colector o saco venoso pulmonar retropericárdico, en el que confluyen las venas pulmonares de ambos pulmones, y que a su vez drena a través de otra vena denominada «vena vertical» a una vena sistémica tributaria de la aurícula derecha. Según el lugar a dónde llegue la vena de drenaje se clasifican en supracardiacos, que son los más frecuentes; cardíacos, infracardiacos y formas mixtas, las más infrecuentes ^5^.

La corrección de la conexión anómala de total de venas pulmonares (CVAPT) supracardiaco consiste en conectar las venas pulmonares a la aurícula izquierda, además de ligar la VV; sin embargo, algunos cirujanos suelen dejar la VV sin ligar para reducir la presión pulmonar, con el objeto de disminuir la crisis hipertensiva en el posoperatorio y favorecer la estabilidad hemodinámica posoperatoria ^5^.

En la mayoría de las ocasiones, la VV se cierra espontáneamente después de la cirugía, debido al flujo preferencial al atrio izquierdo e incremento de su *compliance*. Sin embargo, hay ocasiones donde la VV no ligada o parcialmente ligada permanece patente, lo cual lleva a un cortocircuito de izquierda a derecha ^5^^,^^6^. Kumar refiere que toda VV debe cerrarse ya sea por vía quirúrgica o percutánea, sumado a otros investigadores que recomiendan el cierre de la VV en todas las cirugías de reparación de CVAPT supracardiaca ^4^^,^^7^.

Los primeros reportes del cierre percutáneo de VV se publicaron en 1992, Hausdorf usó un dispositivo para cierre de conducto arterioso para dicho fin, posterior a eso se han venido realizando procedimientos similares con diferentes dispositivos oclusión vascular (Vascular Plug I y Vascular Plug II) ^7^^-^^9^. En nuestro caso no fue posible contar con otro tipo de dispositivo por lo que se utilizó el dispositivo oclusor de comunicación interauricular, lo cual constituye uno de los primeros casos publicados.

En conclusión, la intervención percutánea puede ser considerada como parte de la estrategia de la oclusión de VV luego de la cirugía de reparación de CVAPT supracardiaca con vena vertical permeable. En nuestro caso, el cierre percutáneo de la vena vertical fue realizada con éxito con un dispositivo oclusor de defecto septal interatrial ya que no se contó con otro tipo de material.

## Fuente de financiamiento:

Autofinanciado
